# Construction and Advanced Utilization of Self-Assembled and Scale-Down Chitin Nanofibers for Polymer Composite Design

**DOI:** 10.3390/molecules31020364

**Published:** 2026-01-20

**Authors:** Masayasu Totani, Jun-ichi Kadokawa

**Affiliations:** Graduate School of Science and Engineering, Kagoshima University, 1-21-40 Korimoto, Kagoshima 890-0065, Japan; m-totani@cb.kagoshima-u.ac.jp

**Keywords:** all-chitin composite, cell adhesion, chitin, composite, ionic liquid, nanofiber, nanoparticle, mechanical property, scaffold

## Abstract

This review provides a comprehensive overview of recent progress in chitin-based nanomaterials and their composite engineering. Particular focus is placed on techniques for constructing self-assembled chitin nanofibers (ChNFs) with tightly bundled fibrillar structures, as well as strategies for fabricating composites in which the ChNFs serve as reinforcing components, combined with natural polymeric matrices. In addition, high-crystalline scaled-down (SD-)ChNFs were fabricated through partial deacetylation of the ChNFs, followed by electrostatic repulsive disassembly of the abovementioned bundled fibrils in aqueous acetic acid, which were further used to reinforce composites comprising the other polysaccharides. Mixing the SD-ChNFs with low-crystalline chitin substrates further enabled the fabrication of all-chitin composites (AChCs) that exploit crystallinity contrast to achieve enhanced tensile strength. Moreover, the AChC films exhibited high cell-adhesive properties and promoted the formation of three-dimensional cell-networks, highlighting their potential for biomedical applications.

## 1. Introduction

**Figure 1:**
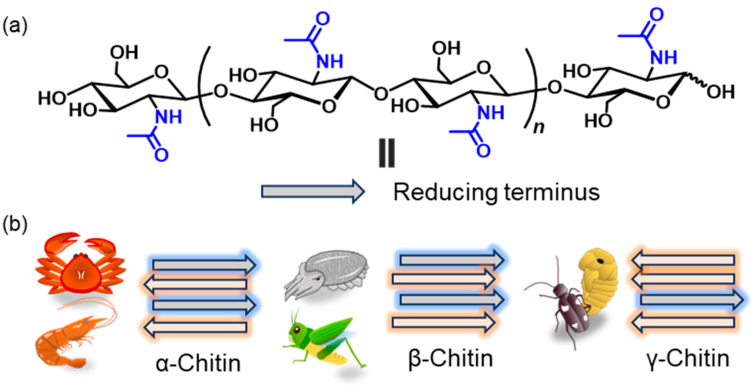
(**a**) Chemical structure of chitin and (**b**) schematic images for representative crystalline structure, α-, β-, and γ-chitins.

**Figure 2:**
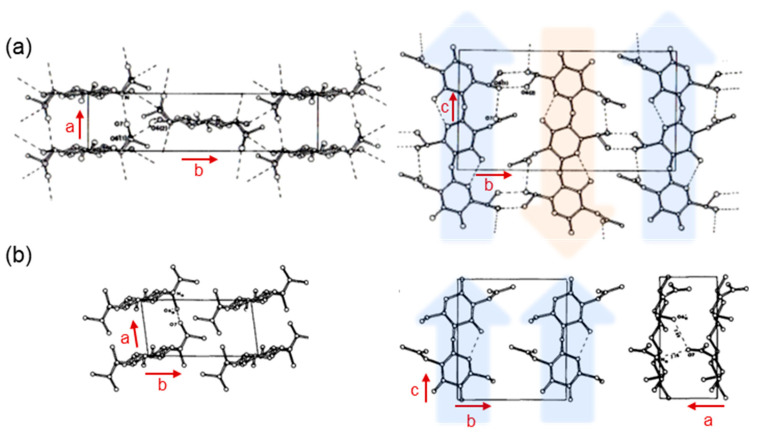
Projection of the proposed model for (**a**) α-chitin (adapted with permission from Ref. [15]. Copyright 1978, Elsevier) and (**b**) β-chitin (adapted with permission from Ref. [14]. Copyright 1975, Wiley).

Chitin is one of the most abundant structural polysaccharides in nature and is mainly biosynthesized in the exoskeletons of crustaceans and insects such as shrimps, crabs, and crickets [1,2,3,4,5,6,7,8,9]. It is a linear polysaccharide composed of *N*-acetyl-D-glucosamine (GlcNAc) units linked through β(1→4) glycosidic bonds (Figure 1a) and is known to exhibit a broad molecular weight on the order of ~10^4^ to ~10^5^ [10,11]. In addition, acetamido groups at C-2 position in the GlcNAc units form strong intermolecular hydrogen bonds, which contribute to the characteristically high crystallinity and stiffness of chitin [12]. The crystalline polymorphs of chitin are classified into three forms (α, β, and γ) according to the chain arrangement (Figure 1b). The most abundant form is α-chitin, which is found in the shells of crabs and shrimps and features antiparallel chain alignment. β-Chitin occurs in the squid pen and cuttlebone of common cuttlefish, as well as in certain insect-derived materials. γ-Chitin, a relatively rare polymorph exhibiting a mixed arrangement of α- and β-type packing, has been reported in the cocoon fibers of *Ptinus* beetles [13]. As a more sophisticated structural representation, the *ab* and *bc* projections of α-chitin and β-Chitin are illustrated in Figure 2a,b [14,15]. The α-chitin lattice exhibits exceptionally tight molecular packing because an extensive alternating array of interchain hydrogen bonds provides a highly stabilized crystalline framework. In contrast, the β-chitin polymorph forms fewer hydrogen bonds, and their geometrical arrangement is less favorable for cohesive packing. As a result, the intermolecular interactions in β-chitin are substantially weaker. Molecular dynamics (MD) simulations, conducted in aqueous environments, have demonstrated that, unlike the robust and structurally persistent α-chitin, the crystalline order of β-chitin undergoes rapid hydration-induced destabilization and readily collapses [16].

Chitin have garnered considerable attention across various research fields owing to their ability to form nanoscale materials such as nanofibers [17,18]. Because of their outstanding characteristics, including light weight, high tensile strength, and low thermal expansion coefficients, nano-fibrillated derivatives such as nanofibers, nanocrystals, and nanowhiskers have been extensively explored as versatile, stable, and functional forms of chitin [19,20,21,22,23,24,25]. Two principal strategies have been reported for the construction of chitin nanofibers, i.e., top-down and bottom-up approaches. The top-down approach involves the disintegration of native chitin microfibrils through mechanical or chemical treatments [18,26,27,28,29,30,31]. Native chitin has a hierarchical structure composed of bundles assembled from nanofibers (microfibrils), and accordingly, size reduction can be achieved by disintegration of the assemblies [32]. This approach generally yields chitin nanofibers with widths on the order of 10 nm and lengths of several micrometers or longer, while some variability in fiber morphology is commonly observed. In contrast, the bottom-up approach relies on dissolving/swelling chitin to temporarily disrupt its crystalline structure, followed by a regeneration process using poor solvents or electrospinning, leading to reconstructing fibrous morphologies. The bottom-up approach enables the facile formation of nanostructures with a wide range of sizes, morphologies, and fiber lengths, spanning from fine particles to fibrous nanofibers. As discussed later, it allows the size of small particles with diameters ranging from several nanometers to several hundred nanometers to be tuned [33,34,35,36].

**Figure 3:**
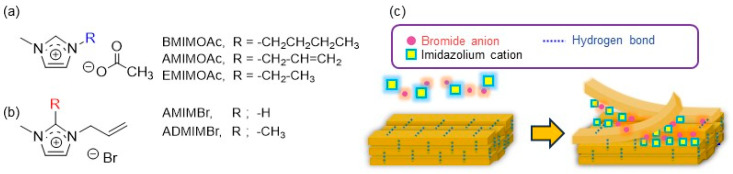
(**a**,**b**) Representative ionic liquids (ILs) capable of dissolving chitin. (**c**) Plausible dissolution process of chitin in AMIMBr predicted by molecular dynamics (MD) simulations.

Ionic liquids (ILs) comprise a family of salts that exhibit liquid-phase behavior near ambient temperatures, where a number of such solvent systems share similar structural and physicochemical features. Rogers et al. demonstrated in 2002 that an IL, such as 1-butyl-3-methylimidazolium chloride (BMIMCl), efficiently dissolved cellulose, which subsequently positioned ILs as highly effective solvents for polysaccharides [37,38,39,40,41]. ILs have also been utilized as effective media for preparing chitin nanofibers (ChNFs) via a regenerative self-assembling process upon bottom-up approach, and thereafter, with the rapid expansion of research in this field, they have further been recognized as one of solvent systems capable of dissolving chitin [42,43,44,45,46]. 1-Butyl-3-methylimidazolium acetate (BMIMOAc) enabled dissolution of chitin from crab shells up to approximately 6 wt%, and the resulting solutions could be subsequently reformed into chitin-sponge architectures [47]. Since then, systematic screening of structurally distinct IL families has led to the reporting of ionic liquids such as 1-allyl-3-methylimidazolium acetate (AMIMOAc) [42] and 1-ethyl-3-methylimidazolium acetate (EMIMOAc) (Figure 3a) [48]. Overall, the above dissolution studies of chitin in ILs suggest that ILs with carboxylate anions generally act as good solvents for chitin.

**Figure 4:**
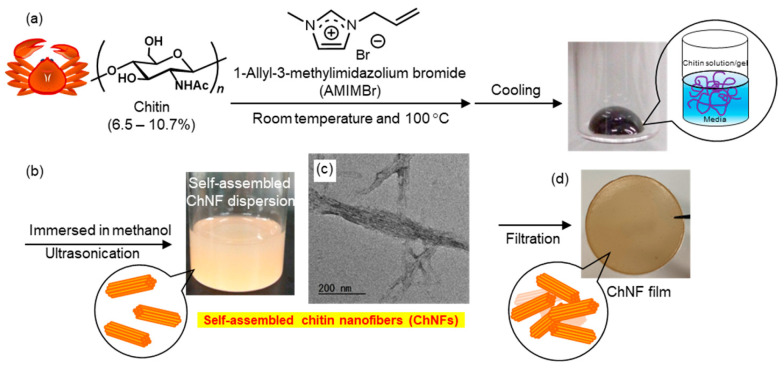
Schematic representation of (**a**) chitin/1-allyl-3-methylimidazolium bromide (AMIMBr) ion gel formation and (**b**) subsequent self-assembly to form chitin nanofibers (ChNFs), (**c**) a representative transmission electron microscopy (TEM) image of the ChNFs, and (**d**) the resulting continuous film (adapted with permission from Ref. [55]. Copyright 2021, Elsevier).

Another type of IL, reported in our previous work, 1-allyl-3-methylimidazolium bromide (AMIMBr) was found to dissolve chitin up to 4.8 wt% [49,50]. Furthermore, when higher amounts of chitin (6.5–10.7 wt%) were mixed with AMIMBr, ion gels were formed after standing at room temperature, followed by heating at 100 °C and cooling [51] (Figure 3b and Figure 4a). To understand the dissolution mechanisms in the ILs, particularly in AMIMBr, MD simulations have provided mechanistic insight into how ILs influence the disintegration of chitin crystallites [52]. The simulations have indicated that bromide ions in AMIMBr efficiently disrupt hydrogen bonds associated with the acetamido groups, thereby promoting progressive chain liberation from the outermost crystalline layers. Once the chains are released, AMIM^+^ act to suppress re-aggregation into an ordered phase (Figure 3c). Recently, it has also been demonstrated that 1-allyl-2,3-dimethylimidazolium bromide (ADMIMBr), a structural analog of imidazolium-based ILs, serves as an efficient chitin solvent [53].

Based on the demonstrated dissolution of chitin in AMIMBr, our laboratory has been developing a fabrication strategy for ChNFs and exploring their translation into advanced functional nanomaterials. The regeneration from chitin/AMIMBr ion gels using methanol with ultrasonication yielded a dispersion of self-assembled ChNFs upon bottom-up process (Figure 4b) [49,54,55,56]. The resulting dispersions were subjected to filtration to produce ChNF films with highly entangled nanofibrous morphologies (Figure 4d). Transmission electron microscopy (TEM) revealed that the ChNFs were composed of bundles of thinner fibrils averaging 20–60 nm in width and several hundred nanometers in length [57] (Figure 4c).

**Figure 5:**
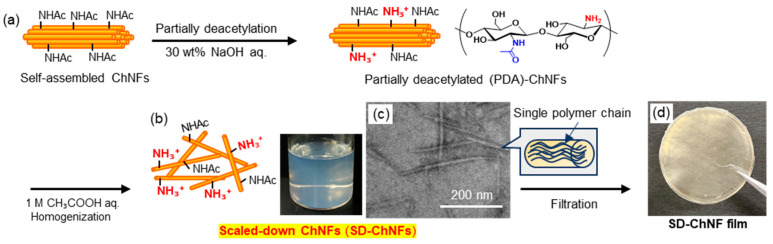
Procedures for the preparation of (**a**) partially deacetylated (PDA)-ChNFs and (**b**) subsequent scaled-down ChNFs (SD-ChNFs), (**c**) a representative TEM image of the SD-ChNFs, and (**d**) the resulting continuous SD-ChNFs film (adapted with permission from Ref. [55]. Copyright 2021, Elsevier).

Partial deacetylation (PDA) of the acetamido groups on ChNFs using 30 wt% aqueous NaOH introduced amino functionalities along the nanofiber surfaces (Figure 5a). Subsequent dispersion of the PDA-ChNFs in 1 M aqueous acetic acid, assisted by homogenization, promoted the formation of individualized, scaled-down (SD-)ChNFs through electrostatic repulsion among the protonated amino (ammonium) groups (Figure 5b). As a result, thinner nanofiber structure was observed in the TEM image (Figure 5c), which was completely different from the bundled morphology observed for the original ChNFs (Figure 4c). The resulting SD-ChNFs were then collected by suction filtration to produce flexible films with densely entangled morphologies (Figure 5d).

The obtained self-assembled and SD-ChNFs are solely composed of chitin and considered to possess intrinsic biocompatibility, biodegradability, and mechanical robustness. Owing to these properties, they are expected to serve as sustainable bio-nanomaterials for biomedical scaffolds, film substrates, and environmentally friendly composite systems, where nanoscale organization plays a key role in determining their material performance [18,25,58,59,60,61,62,63,64,65,66,67,68,69,70,71,72,73,74,75,76,77,78,79,80,81,82,83,84,85,86,87,88].

## 2. Fabrication of Polymer Composites Based on Chitin Nanofibers

**Figure 6:**
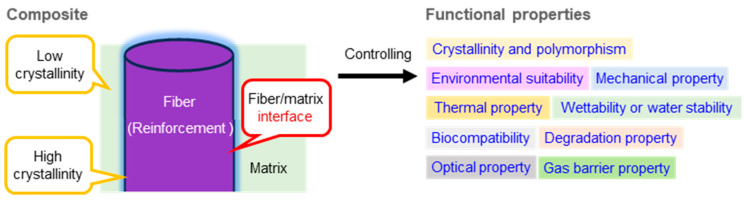
Schematic illustration of a polymer composite structure and its functions.

Composite structures can be constructed from a single component or by combinations of multiple components. In such systems, the interface between the reinforcement phase and the matrix phase should be precisely controlled, which is recognized as a key factor for governing the overall mechanical property of the composite (Figure 6) [79,88,89,90,91,92,93,94,95,96,97,98,99,100,101,102,103,104,105,106,107]. Variations in crystallinity between the fiber and matrix components critically influence interactions and load distribution and consequently modulate a broad spectrum of functional characteristics. These include mechanical performance, thermal and environmental stabilities, wettability and water resistance, optical and gas barrier properties, biocompatibility, and degradation behavior. Thus, the rational design of composite materials necessitates deliberate tuning of component crystallinity together with quantitative engineering of the fiber–matrix interface to achieve targeted structural and functional outcomes. In our research, we have focused on the ChNFs as a reinforcing component, and accordingly, have examined their composition with various polymeric substrates as matrix candidates [108,109,110]. In recent years, we have also investigated systems using natural polymers as the matrixes.

**Figure 7:**
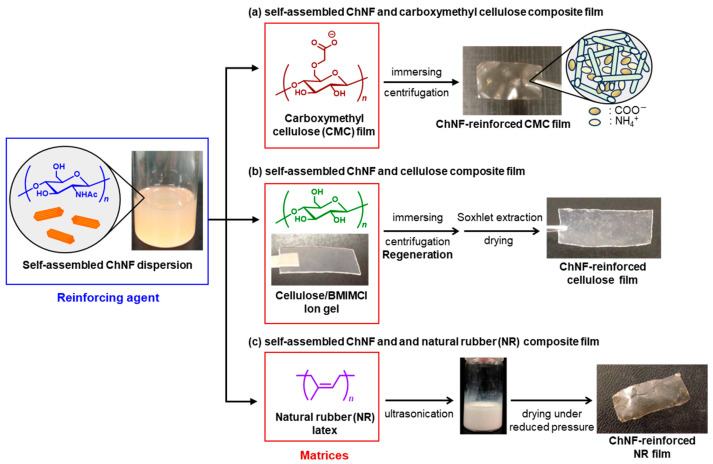
Schematic illustration of preparations of (**a**) self-assembled ChNF/carboxymethyl cellulose (CMC) composite, (**b**) self-assembled ChNF/cellulose composite, and (**c**) self-assembled ChNF/natural rubber (NR) composite.

As an example, we established a simple method to fabricate ChNF-reinforced carboxymethyl cellulose (CMC) films through electrostatic interaction between ammonium and carboxylate groups (Figure 7a) [111]. CMC films were immersed in self-assembled ChNF dispersions prepared by regeneration from the chitin/AMIMBr ion gels. The absorbed ChNF amount in the composites increased with ChNF content in the dispersions, as confirmed by SEM and IR analyses. Tensile testing revealed that the ChNFs effectively reinforced the CMC films to enhance mechanical strength. The results indicate that the self-assembled ChNFs serve as promising components for compatibilizing with acidic polysaccharides, offering a versatile route to develop practical bio-based composite materials.

In another approach, the self-assembled ChNF-reinforced cellulose films were prepared via stepwise regenerations from the two ion gels (Figure 7b) [112]. The ChNF dispersion was obtained by the abovementioned regeneration process from the chitin/AMIMBr ion gel using methanol. In parallel, a cellulose/BMIMCl ion gel was prepared and immersed in the ChNF dispersion, allowing simultaneous regeneration of cellulose nanofibers and formation of a ChNF/cellulose composite film. IR and SEM analyses confirmed distribution of the ChNFs throughout the film. The X-ray diffraction (XRD) result revealed the amorphous structure of the regenerated cellulose component in the composite film. Tensile testing showed that the ChNFs significantly enhanced the mechanical strength. The regeneration-based approach offers a versatile route for fabricating well compatible, ChNF-reinforced polysaccharide materials applicable to various natural biopolymers.

Furthermore, the self-assembled ChNF film was found to be dispersible in ammonia aqueous solution. In addition, natural rubber (NR) is known to form an anionically charged colloidal latex with ammonia aqueous solution. Accordingly, ChNF/NR latex dispersions were obtained by mixing the two dispersions in different ratios under ultrasonication (Figure 7c) [113]. The resulting dispersions were evaporated in a drying tray under reduced pressure, yielding ChNF/NR composite films, in which the ChNFs acted as a reinforcing component, as an increase in the ChNF content strengthened the composite films. On the other hand, heating the ChNF/NR mixture for removal of ammonia, followed by lyophilization, yielded porous composite materials. The formation of the porous structures was attributed to aggregation of the ChNFs and NR upon loss of ammonia and the subsequent generation of void spaces between aggregated domains during the lyophilization process.

**Figure 8:**
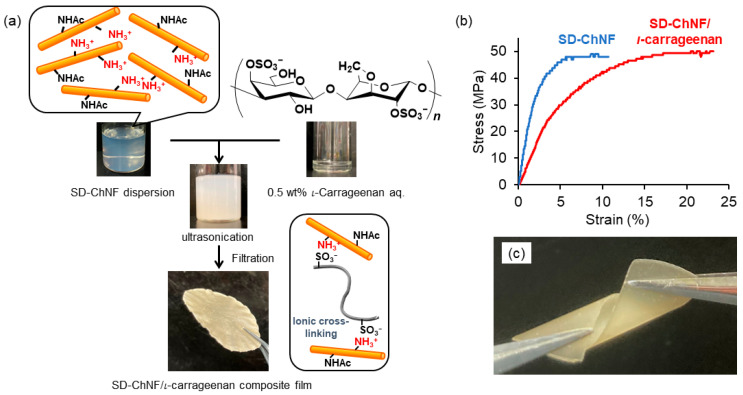
Schematic illustration of (**a**) preparation of SD-ChNF/ι-carrageenan composite film with dispersion solutions, (**b**) stress–strain curves of SD-ChNF and composite films, and (**c**) photograph for twisting test of composite film.

To further impart functional properties to the SD-ChNF film, we investigated the fabrication of a composite with a ι-carrageenan film by electrostatic interaction as exploited for CMC (Figure 8a) [55]. ι-Carrageenan is a water-soluble phycocolloid derived from red algae, composed of alternating 1,3-linked α-D-galactopyranose and 1,4-linked β-3,6-anhydro-D-galactopyranose residues bearing sulfate groups at the C-4 and C-2 positions [114]. A dispersion of the ChNFs in aqueous acetic acid was added dropwise onto a viscous 0.5 wt% ι-carrageenan aqueous solution (NH_3_^+^:SO_3_^−^ feed ratio = 1:1.2). The resulting mixture was ultrasonicated for 10 min and then filtered to yield the composite film. Based on the film weight, the NH_3_^+^:SO_3_^−^ ratio was calculated to be 1:0.76, indicating that the composite was efficiently formed from two components bearing positive and negative charges. The stress–strain curve of the SD-ChNF/ι-carrageenan composite film under tensile mode exhibited a markedly improved elongation at break while maintaining a tensile strength comparable to that of the neat SD-ChNF film (Figure 8b). The twisting test further revealed the flexible yet brittle nature of the composite film (Figure 7c). These results suggest that combining the SD-ChNFs with anionic polysaccharides facilitates the formation of efficient ion-pair interaction, which in turn contributes to the overall mechanical strength of the composite film.

## 3. Concept and Design of All-Chitin Composites Based on Single-Polymer Composite Principle

**Figure 9:**
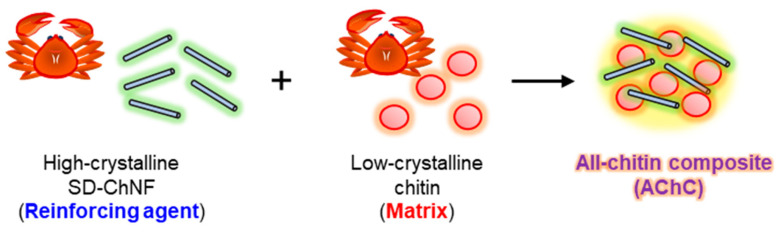
Schematic illustration for concept of all-chitin composite (AChC).

Single-polymer composites (SPCs) are defined as composed entirely of a single polymer [115,116]. Their properties are governed by intermolecular interactions, the balance of amorphous and crystalline regions, and hydrogen bonding networks, which distinguish them from conventional multi-component composites. Moreover, their thermal stability and degradation products can be readily predicted [18,117]. To translate the SPC concept into a chitin-based system, we have hypothesized that all-chitin composites (AChCs) are constructed by deliberately exploiting crystallinity contrast within a single polymer. In this design concept, highly crystalline SD-ChNFs would serve as the reinforcing phase, whereas low-crystalline chitin would serve as the matrix. We further postulated that integrating these structurally distinct yet chemically identical components would create a self-reinforced composite framework, in which mechanical reinforcement, stress transfer, and functional tunability arise solely from the controlled hierarchical organization of chitin itself (Figure 9).

### 3.1. Fabrication of AChCs Using Low-Crystalline SD-ChNFs Prepared via Trifluoroacetic Acid Treatment

**Figure 10:**
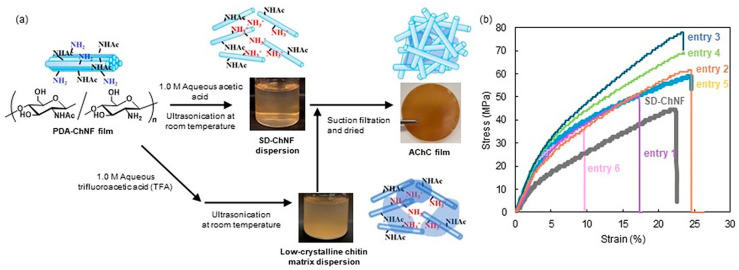
Schematic illustration of (**a**) preparation of AChC with SD-ChNF dispersion with 1.0 M in aqueous acetic acid and low-crystalline chitin matrix dispersion with 1.0 M aqueous trifluoroacetic acid (TFA), (**b**) stress–strain curves of the SD-ChNF film and AChC films with varying low-crystallinity chitin/SD-ChNF weight ratios: entry 1 (0.006/1), entry 2 (0.013/1), entry 3 (0.026/1), entry 4 (0.067/1), entry 5 (0.091/1), and entry 6 (0.143/1) under tensile mode (adapted with permission from Ref. [119], Copyright 2025, Elsevier).

We first attempted to fabricate AChCs by blending highly crystalline SD-ChNFs with a low-crystalline chitin substrate derived from the PDA-ChNFs, based on a double-crystalline concept in which two chitin components with distinct crystallinities are combined (Figure 10a) [110,118,119]. To obtain a low-crystalline chitin matrix, the PDA-ChNFs were ultrasonicated in several 1M aqueous acid solutions. The XRD and IR analyses showed that acetic and formic acids preserved nanofiber morphology and maintained high crystallinity, whereas mild heating and ultrasonication in trifluoroacetic acid (TFA) effectively reduced crystallinity to a crystalline index (CI) of approximately 82%, without inducing chemical degradation. In contrast, methanesulfonic acid caused significant decomposition and was therefore excluded from further investigation. These low-crystalline chitin dispersions by TFA treatment (1M aqueous solution) were subsequently mixed with the SD-ChNF/1M aqueous acetic acid dispersions at various weight ratios, followed by filtration and drying, to prepare AChC films, as shown in Figure 10a. Increasing the proportion of the low-crystalline matrix gradually decreased the overall crystallinity of the resulting films. Tensile testing revealed a clear dependence of mechanical properties on composite composition. In this series, entries 1 through 6 correspond to the AChC films prepared with progressively increasing proportions of the low-crystalline chitin matrix, allowing systematic assessment of how matrix content influences mechanical reinforcement (entries 1, 2, and 3) (Figure 10b). Young’s modulus and tensile strength increased with the addition of the low-crystalline matrix up to an optimal SD-ChNF to matrix ratio of 1 to 0.026 (entry 3), where the composite achieved a Young’s modulus of 0.763 GPa and a tensile strength of 78 MPa, while maintaining an elongation at break similar to that of the pure SD-ChNF film. Further increases in the matrix content (entries 4 and 5) reduced reinforcement efficiency, presumably because of insufficient stress transfer at the fiber to matrix interface.

Overall, these results demonstrate that precise control of crystalline among chemically identical chitin-based components provides a useful strategy for introducing complementary mechanical functions into all-chitin composites without the need for external polymers.

### 3.2. Construction of Low-Crystalline Chitin Nanoparticles and Their Application as Matrix Components in AChCs

**Figure 11:**
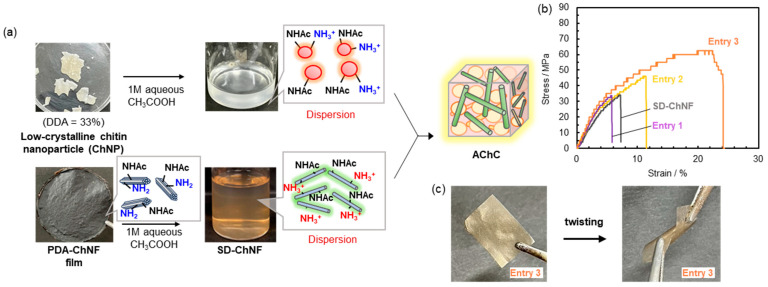
(**a**) Schematic illustration for preparation of self-reinforced AChCs from low-crystalline chitin nanoparticle (ChNP) and SD-ChNF dispersions, (**b**) stress–strain curves of AChC films with varying SD-ChNF/ChNP weight ratios (*w*/*w*); entry 1 (1/0.028), entry 2 (1/1), and entry 3 (1/6.6), and (**c**) photographs for twisting test of AChC film (entry 3) (adapted with permission from Ref. [110]. Copyright 2025, Elsevier).

TFA has been employed in the abovementioned study because it is widely used in bio-related research as an effective solvent for weakening hydrogen-bonding interactions. However, its inherent toxicity poses limitations based on the viewpoint of practical application. In addition, the crystallinity of chitin obtained through the TFA treatment was not sufficiently reduced as low-crystalline chitin components. Therefore, in the following study, we aimed to construct low-crystalline chitin nanoparticles via an alkaline treatment in order to prepare a chitin matrix with lower crystallinity than that obtained through the previous studies based on the TFA treatment (Figure 11a). Chitin with relatively reduced crystallinity is known to be obtainable by partial deacetylation through NaOH treatment in aqueous media [110]. Additional deacetylation of the resulting low-crystalline chitin raised the degree of deacetylation (DDA) to 33%. Accordingly, the powdered chitin substrate with nanoparticle (ChNP) morphology was obtained, which dispersed well in aqueous acetic acid [120]. The SEM images revealed the following clear morphological differences; the SD-ChNFs formed nano fibrous structure, whereas the ChNPs existed as ca. 10 nm spherical particles. The XRD analysis confirmed their difference in crystallinity, where the SD-ChNF film exhibited CI over 90.5%, while the ChNPs showed CI of 57.4%, which was significantly lower than that of the TFA-treated chitin substrate. The AChC films were prepared by mixing the SD-ChNF and ChNP/1M aqueous acetic acid dispersions at defined ChNF/ChNP weight ratios (entry 1, 1/0.028; entry 2, 1/1; entry 3, 1/6.6), followed by filtration (Figure 11a). The CI values of the resulting AChC films were found to be tunable, as 65.4–88.5%, by adjusting the ChNF/ChNP weight ratios [110,121]. Increase of ChNP contents in the AChC films gradually obscured and lowered crystallinity, consistent with integration of amorphous domains. Moreover, laser microscopy showed that the SD-ChNF film displayed rough surfaces, while the AChC films became smoother with increasing ChNP contents although root-mean square roughness values did not vary significantly (0.97–2.10 µm).

Tensile testing of the AChC films revealed a clear composition-dependent trend, in which increasing the ChNP content enhanced tensile strength and Young’s modulus (Figure 11b). At the optimal composition (SD-ChNF to ChNP ratio of 1 to 6.6, entry 3), the composite exhibited markedly improved mechanical performance, reaching 64.0 MPa in tensile strength and 0.924 GPa in Young’s modulus. In contrast, films with insufficient ChNP content displayed limited reinforcement. Entry 1 showed values of 33.8 MPa (strength) and 0.742 GPa (modulus), while entry 2 exhibited 47.5 MPa and 0.769 GPa. These values were comparable to or only marginally higher than those of the reference SD-ChNF film (34.0 MPa and 0.608 GPa). The inferior performance at low-ChNP contents underscores the brittleness associated with ChNF rich compositions (entries 1 and 2) and highlights the necessity of establishing an appropriate balance between the high-crystalline SD-ChNFs and low-crystalline ChNPs. The IR analysis supported strong interfacial hydrogen bonding between acetamido and hydroxy groups, which facilitated stress transfer within the composite.

Collectively, these findings demonstrate that controlled blending of chitin components with distinct crystallinities offers an effective means to tune the mechanical response of the AChCs, and notably, that the optimally composed film in entry 3 achieves a level of structural integrity sufficient to withstand twisting deformation without fracture, thereby highlighting the robustness of the resulting AChC architecture (Figure 11c).

### 3.3. Hydration and Cell Adhesion Properties on AChC Films

**Figure 12:**
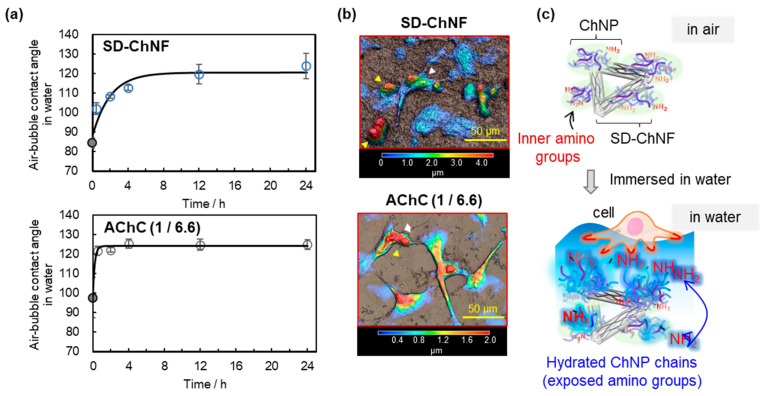
(**a**) Air bubble contact angles against SD-ChNF and AChC (SD-ChNF/ChNP 1/6.6 wt/wt) films, (**b**) laser microscope images of HeLa cell adhesion on SD-ChNF and AChC films, and (**c**) hypothesis illustrations of AChC film surface in air to water environments (adapted with permission from Ref. [121]. Copyright 2025, Elsevier).

We have investigated how the hydration behavior of the AChC film composed of the highly crystalline SD-ChNFs and low-crystalline ChNPs at an affixed mixing ratio of 1 to 6.6 (wt/wt) [110,121]. Hydrophilicity was evaluated by measuring air bubble contact angles in aqueous media. In the dry state, the AChC film showed a slightly higher theoretical contact angle than the pure SD-ChNF film, reflecting the increased ChNP content and its contribution to hydrophilicity. Upon water immersion, the AChC film underwent a more rapid transition toward a hydrated, hydrophilic surface compared to the SD-ChNF film, consistent with the faster reorientation of hydrophilic amino rich ChNP domains at the interface. After 24 h of immersion, however, both films converged to similar values of approximately 120–125° (Figure 12a).

Cell adhesion studies using HeLa cells revealed composition-dependent responses. The SD-ChNF film supported very limited adhesion, with most HeLa cells remaining as small spherical aggregates. In contrast, the AChC film (SD ChNF/ChNP = 1/6.6 wt/wt) exhibited markedly enhanced cell adhesion, indicating that the optimized combination of highly crystalline nanofibers and low-crystalline chitin nanoparticles provides a surface environment considerably more favorable for cell adhesion (Figure 12b). The results support that hydration-induced exposure of cationic amino groups from the ChNPs at the interface enhances electrostatic interactions with negatively charged cell membranes (Figure 12c). Strong adhesion of the AChC film with high-ChNP content thus likely stems from synergy between structural support by the SD-ChNFs and hydrophilic amino-rich ChNP domains exposed in aqueous environments.

### 3.4. High-Density Cell Adhesion Behavior for Three-Dimensional Scaffold

**Figure 13:**
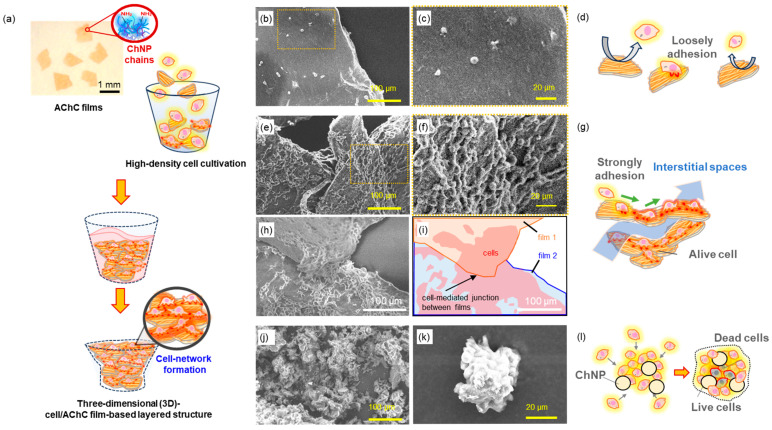
(**a**) Schematic illustrations of three-dimensional (3D-)cell/AChC film-based layered structure with high-density cell cultivation, SEM images and schematic illustration of adherent HeLa cells on (**b**–**d**) SD-ChNF films, (**e**–**i**) AChC films, and (**j**–**l**) ChNP powders.

Based on the above experimental results, we hypothesized that the highly cell-adhesive AChC films could be applied to tissue-like three-dimensional (3D-)cell culture systems [122,123,124,125,126,127,128,129], such high-density culture on the AChC films would induce network-like cell adhesion that connects multiple films into a layered 3D-cell/film structure (Figure 13a) [130]. The AChC films were added to a HeLa suspension (3 × 10^6^ cells mL^−1^), and the SD-ChNF films and ChNP powders were also tested as references. After seeding, inversion mixing was performed every hour for 6 h, and after 4 days of static culture, cells were fixed. Sterilization tolerance indicated that the SD-ChNF and AChC films maintained flat surfaces after autoclaving at 121 °C for 20 min, whereas the ChNPs remained as 50 nm aggregates without redispersion. Thus, both film types showed significant aqueous stability owing to strong intermolecular hydrogen bonding. The SEM measurement revealed that the SD-ChNF films supported only sparse, weakly adherent spherical cells (Figure 13b–d). In contrast, the AChC film promoted pronounced cellular elongation and the formation of multicellular networks that bridged adjacent film fragments, thereby generating 3D connectivity. The overlapping arrangement of the AChC film fragments also created interstitial spaces, which likely served as pathways for fresh culture medium to permeate the structure and support sustained cellular viability (Figure 13e–i)). In contrast, the ChNP powders failed to sustain robust adhesion and instead induced only small, compact spheroids (Figure 13i,k). Such tightly aggregated cell spheroid raises concerns that, during prolonged culture, cells located in the central region may not receive sufficient nutrient and oxygen supply, potentially leading to progressive cell death within the core (Figure 13l).

Therefore, the AChC films outperform both the SD-ChNF films and ChNP powders as scaffolds for 3D cell culture. The optimized combination of the SD-ChNFs and ChNPs yields the film with mechanical integrity and high-cell-interaction ability. This suggests a strategy of stacking the AChC film fragments to construct 3D architectures with sufficient interstitial space for nutrient and oxygen diffusion, unlike spheroid-based approaches that often suffer from diffusion limitations.

## 4. Conclusions

This review outlines approaches for constructing composite materials by combining the self-assembled ChNFs with various natural polymers. It also demonstrates that the SD-ChNFs, which retain high crystallinity and are finely dispersed, can be incorporated into these systems and blended with natural polymers. Furthermore, combining the SD-ChNFs with low-crystalline chitin substrates yielded the AChCs that embody the single-polymer composite concept. These AChCs exhibited experimentally verified improvements in mechanical performance, hydration properties, and cell adhesion behavior, confirming the effectiveness of crystallinity contrast in controlling composite functionality. Future studies will focus on detailed toxicological evaluations and systematic investigations on cellular responses to facilitate further development toward medical applications. Furthermore, these fundamental insights into material properties and biosafety provide important guidelines for future industrial implementation and indicate potential applicability to large-scale structural and transportation-related sectors requiring high material volumes. Collectively, chitin nanofibers provide a coherent and experimentally validated framework for the development of sustainable nanomaterials and advanced composites for materials science and bioengineering applications.

## Data Availability

The data presented in this study are available on request from the corresponding author.
